# Social Metamemory Judgments in the Legal Context: Examining Judgments About the Memory of Others

**DOI:** 10.3390/bs15070878

**Published:** 2025-06-27

**Authors:** Rebecca K. Helm, Yan Chen

**Affiliations:** Law School, Faculty of Humanities and Social Sciences, University of Exeter, Stocker Road, Exeter EX44QJ, Devon, UK; y.chen13@exeter.ac.uk

**Keywords:** social metamemory, cognitive science and law, juror decision making, eyewitness identification, corpus linguistic analysis

## Abstract

Jurors and other legal decision-makers are often required to make judgments about the likely memory accuracy of another person. Legal systems tend to presume that decision-makers are well-placed to make such judgments (at least in the majority of cases) as a result of their own experiences with memory. However, existing research highlights weaknesses in our abilities to assess the memories of others and suggests that these weaknesses are not easily ameliorated through the provision of information. In this work we examine the accuracy of layperson assessments of “real” eyewitness identifications following observation of a mock crime. We examine whether novel instructions, characteristics and beliefs of assessors, and underlying reasoning strategies are associated with improved or impaired judgment accuracy. The results support prior research in demonstrating a tendency towards over-belief in the accuracy of identifications. They suggest that reliance on what witnesses have said rather than attempts to make inferences from their statements (e.g., in relation to the level of detail provided or non-verbal cues in testimony) is associated with greater accuracy in assessments and that some individual differences and beliefs about memory are also associated with greater accuracy. However, there was no evidence that the instructions tested were effective. We discuss the implications of results for procedure surrounding the evaluation of memory in the legal context.

## 1. Introduction

Eyewitness accounts are often important evidence in the legal context, and identification evidence specifically has been described as a ‘staple form of evidence’ used to identify criminal offenders (Wells et al., 2020). As part of their role as legal fact-finders, jurors often have to assess the likely reliability of identification evidence (a form of ‘social’ metamemory judgment). Legal systems often operate under the presumption that laypeople (and therefore jurors) can make these judgments effectively as the result of their own experiences with memory, and therefore leave jurors with the task of making such assessments with relatively little assistance (for a discussion of the way that courts expect jurors to evaluate eyewitness testimony in the US, the UK, and Australia, see Semmler et al., 2012). However, a relatively significant body of work examining lay decision making in this area suggests that actually laypeople are susceptible to perform poorly when making such judgments, particularly through over-confidence in the accuracy of identifications. Incorrect eyewitness identification evidence remains a leading cause of miscarriages of justice across multiple jurisdictions. In May 2025, the US National Registry of Exonerations categorized 995 of the 3676 post-1989 exonerations in their database as having involved a mistaken witness identification (National Registry of Exonerations, n.d. For similar information in relation to other jurisdictions, see Evidence Based Justice Lab Miscarriages of Justice Registry, n.d.; Canadian Registry of Wrongful Convictions, n.d.; European Registry of Exonerations, n.d.; see also Helm, 2021b, 2022; Howe et al., 2017). In many of these miscarriages of justice, jurors were willing to convict a defendant based on eyewitness identification evidence (sometimes with clear flaws) that later turned out to be incorrect. In one Scottish case, the appeals court even came to the “clear conclusion” that the jury (who had convicted the defendant based primarily on identification evidence) had come to a conclusion that “no reasonable jury” could have reached (John Jenkins v Her Majesty’s Advocate, 2011). In this context, it is helpful to closely scrutinize how laypeople make assessments of identifications made by others, how the accuracy of those assessments may vary in different conditions, and how legal infrastructure can promote more effective assessments and, relatedly, more effective administration of justice. In this paper, we examine layperson (assessor) assessments of “real” identifications made by research participants following observation of a mock crime. We examine differences in the reasoning underlying inaccurate and accurate assessments and the influences of instructions and assessor characteristics on assessment accuracy.

### 1.1. Social Metamemory Judgments in the Identification Context

Metamemory judgments have been most extensively studied in the context of judgments of learning. Research in this context suggests that assessments of the memory of others (social metamemory judgments), as with assessments of own memory, are influenced by two broad types of cues—experience-based cues, which come from experience of a stimulus at hand (e.g., Alter & Oppenheimer, 2009), and belief-based cues, which come from beliefs or theories about memory (e.g., Frank & Kuhlmann, 2017). In the context of own memory, when someone is assessing whether they believe that they will remember a face, they may be influenced by how memorable that face seems to them upon viewing it (an experience-based cue) and their beliefs as to the general memorability of faces and how often they tend to remember faces (a belief-based cue). In the context of others’ memory, both of these types of cues are particularly susceptible to inaccuracy (see Helm & Growns, 2023). Experience-based cues involve translating own experience to the likely memory of another who experienced the stimulus differently and may find different things memorable. Belief-based cues involve applying beliefs and intuitions built on experience of own memory to assess the memory of someone else, who may experience memory differently. Therefore, while the ‘intuitions’ relating to memory that are often relied on by legal systems may be informative in assessing own memory (e.g., Wixted & Wells, 2017), they are particularly susceptible to inaccuracy in relation to assessments of the memory of others. It is therefore important to examine the ability of decision-makers to assess the memories of others as well as cues associated with more and less accurate assessments in order to examine the likely ability of jurors in this area and to design intervention if appropriate. Such an examination reveals areas of weakness in the ability of decision-makers to make such judgments.

Research suggests that laypeople’s views about memory are inconsistent with expert opinion based on scientific research (e.g., Benton et al., 2006; Kassin et al., 2001; Simons & Chabris, 2011, 2012). Perhaps most importantly in the legal context, a significant body of evidence shows that people generally over-estimate the reliability of memory. In one US-based study reported in 2011, examining survey responses from 1500 members of the public and 2 expert samples (N = 16 and N = 73) found that, while no experts agreed (strongly or mostly) that “Human memory works like a video camera, accurately recording the events we see and hear so that we can review and inspect them later,” 63% of the public sample strongly agreed or mostly agreed with this statement (Simons & Chabris, 2011; see also Simons & Chabris, 2012 for similar results in an different sample of participants). This research also highlights the potential for laypeople to be misled by particular cues in some contexts. For example, the research suggests that laypeople may be prone to misinterpreting witness confidence. Benton et al. (2006) surveyed 111 jurors (individuals who had responded to a jury summons) and found that only 50% believed that an eyewitness’s confidence can be influenced by factors that are unrelated to identification accuracy (a statement endorsed by 95% of experts in a prior study; see Kassin et al., 2001). Experimental work has confirmed the likely importance of this over-estimation of the accuracy of memory in assessments of identification evidence. That work has typically asked research participants to assess the likely accuracy of an identification given by another person who has witnessed an event (e.g., a mock crime), and has found that people are biased towards believing that identifications are accurate (Helm & Spearing, 2025; Lindsay et al., 2000). Insight into the ability of people to distinguish between accurate and inaccurate identification evidence generally can also be gleaned from this work, although accuracy levels have varied by study. Studies utilizing these paradigms have found assessors to be accurate 63.6% of the time (Martire & Kemp, 2009, using an Australia-based undergraduate psychology participant pool), accurate 73% of the time in assessments of true identifications and 51% of the time in assessments of false identifications (Lindsay et al., 2000, using a US-based undergraduate participant pool), and between 42% and 70% of the time (depending on the race of the target and witness; see Helm & Spearing, 2025, using a UK-based non-student participant pool). Thus, research suggests that while people are potentially picking up on some probative cues, intuitions as to the reliability of identifications are far from reliable themselves, and careful intervention is needed to guide these judgments in the legal process.

Existing research also provides insight into cues that laypeople rely on in assessing the memory of others, providing insight into potential sources of accuracy and inaccuracy in assessments. This research has tended to use controlled mock trial materials and to experimentally manipulate factors of interest in the trial materials. Factors that have been found to make an eyewitness account less believable in this work include a lower degree of detail (Bell & Loftus, 1988), (even minor) inconsistencies (Bruer & Pozzulo, 2014; Fraser et al., 2022; O’Neill & Pozzulo, 2012; Lavis & Brewer, 2017; although see Lindsay et al., 1986, where such an effect was not found), and lower confidence (e.g., Cutler et al., 1990, although the effect size appears to be modest; see Slane & Dodson, 2022) in an identification or account more broadly. However, these cues may be misleading in predictable contexts (on the relationship between level of detail and accuracy, see Weber and Brewer (2008)). Even inconsistencies, which are sometimes viewed as clear indications of either poor memory or deception, are now thought to tell us “little or nothing” about the accuracy of witness testimony, apart from that of the specific inconsistent statement (Fisher et al., 2012, p. 178; see also Hudson et al., 2020). The relationship between confidence and accuracy is more complex. Although highly confident memories can be false (e.g., Loftus & Greenspan, 2017), a recent analysis of research relating to confidence and eyewitness identification accuracy suggests that, at least under ‘pristine’ conditions, confidence is a relatively good predictor of accuracy and high confidence identifications are more likely to be accurate (Wixted & Wells, 2017). That work has provided rough indications (based on averages from 15 studies) of likely accuracy for different bands of confidence (0–20% confidence ≈ 65% correct; 30–40% confidence ≈ 75% correct; 50–60% confidence ≈ 80% correct; 70–80% confidence ≈ 90% correct, 90–100% confidence > 95% correct) (Wixted & Wells, 2017; and note the probability of an identification of a suspect being correct is higher than this since an inaccurate identification could be of a filler). It is therefore possible that confidence could be a helpful cue for people to rely on in assessing eyewitness identification evidence (particularly since even experts have struggled to identify more reliable cues as to memory accuracy, e.g., Bernstein & Loftus, 2009), provided that it is not treated as dispositive and the possibility of confident false memory is considered in each case, as well as the potential for non-pristine conditions underlying the identification.

### 1.2. The Utilization of Instructions

One way to improve assessments of identifications made by others may be to utilize judicial instructions through which relevant information, for example, information on how to utilize confidence as a cue in assessing identification accuracy, is provided to assessors. Instructions are already provided to jurors in a number of jurisdictions; however, these instructions largely focus on providing general warnings to jurors relating to the unreliability of identification evidence (e.g., Telfaire instructions (United States v Telfaire, 1972), Henderson instructions (New Jersey v Henderson, 2011), Turnbull instructions (R v Turnbull, 1977)). A significant number of miscarriages of justice resulting from mistaken identifications have persisted in spite of these warnings, and experimental research suggests that the instructions may be ineffective both in causing assessors to update their beliefs (Helm, 2021a) and in influencing their decision making (Helm, 2021a; Dillon et al., 2017; Jones et al., 2017). Some research provides insight into why these instructions might be ineffective. For example, some research suggests that the instructions communicate information to jurors in a verbatim form that they struggle to engage with, and that instructions may be improved by making content more *meaningful* to jurors, allowing them to apply the instructions in a concrete way in practice (Helm, 2021a). In line with this idea, the instructions that have been shown to be effective in increasing sensitivity (rather than just skepticism) in experimental work utilizing scripted materials have all focused on educating or training potential jurors rather than providing general warnings or information (Jones & Penrod, 2018; Ramirez et al., 1996; Jones et al., 2020; Helm, 2021a).

Existing experimental research found that providing information relating to witness confidence to assessors (specifically in the form of expert evidence either describing confidence as a good indicator of accuracy or stating that there is no relationship between confidence and accuracy) had no appreciable effect on the accuracy of assessments of identifications made by witnesses to a mock crime (Martire & Kemp, 2009). However, in line with the research discussed above, providing this information in a more meaningful way that can be applied in practice may be more effective. One way to make information relating to confidence meaningful for jurors may be to provide meaningful quantitative benchmarks to assist them in using confidence as a cue to assess accuracy (without overriding consideration of other cues). Research in other contexts has shown that meaningful anchors can help to orient (rather than bias) juror decisions (see, for e.g., Hans et al., 2022). The rough benchmarks identified by Wixted and Wells (2017) may be helpful in this regard in guiding jurors through the general utility of confidence, alongside warning them to consider other contextual factors that may impact the reliability of an identification. In this work, we examine the impact of both traditional instructions warning assessors that a confident identification may be wrong and instructions incorporating benchmarks from Wixted and Wells (2017) on the way that assessors interpret confidence, and on the accuracy of assessor judgments in relation to identification evidence.

### 1.3. Assessor Characteristics and Beliefs

A less explored area in assessing factors that may promote the accuracy of assessments of identification evidence is examining characteristics and beliefs of assessors themselves that have the potential to make them better or worse at making social metamemory judgments. Although this information may be less easy to utilize directly to improve legal systems, it provides new insight into the nature of assessments of the memory of others, including into psychological processes underlying more accurate and less accurate judgment, and therefore can be drawn on in order to identify ways in which such assessments may be improved (either via juror selection or via interventions to target factors that make particular individuals prone to poorer assessments). In this work, we consider four sets of characteristics and beliefs of assessors that may influence their assessments of the memory of others: experiences with memory, beliefs about memory, gist vs. verbatim processing, and beliefs about the criminal justice system.

First, assessors’ own experiences of memory (e.g., as reliable or unreliable) may influence what they expect from a witness, with those with experience of reliable memory being more likely to classify a memory as true (and therefore being more accurate in the identification of correct identifications) and those with experience of unreliable memory being more likely to classify a memory as false (and therefore being more accurate in the identification of false identifications).

Second, people’s beliefs about memory (and about the cues that are probative in assessing memory) may influence the accuracy of identification assessments. People with beliefs about memory that are consistent with evidence and expert opinion may be more effective at assessing the memory of others, and therefore more likely to make accurate judgments in relation to the memory of others.

Third, the way that people process information in decision making, including the tendency to rely on more gist-based processing (focused on meaningful and contextual interpretation of a stimuli) or verbatim-based processing (focused on precise and superficial details of a stimuli; see Reyna, 2012) may impact their effectiveness in evaluating memories of others. Reliance on meaningful information rather than picking up on superficial misleading cues (such as those discussed above) could potentially have a positive effect on assessment accuracy. In this work, we indirectly examine the relationships between people’s cognitive processing and their assessments of the memory of others, utilizing a scale designed to measure autistic traits in the general population. The link between autistic traits and reliance on gist has been discussed in prior work (Reyna & Brainerd, 2011) and stems from the fact that individuals with autism tend to focus on parts rather than “global aspects” of objects and have difficulty integrating information into a meaningful whole (e.g., Frith & Happé, 1994; for discussion, see Reyna & Brainerd, 2011). As a result, researchers have suggested that those with autism (and those showing relevant autistic traits) will experience disadvantages in utilizing gist-based intuition (which requires integrating elements in a meaningful and sometimes abstract way) and advantages in utilizing verbatim analytical processing (which requires attention to precise and sometimes superficial details) (see Reyna & Brainerd, 2011).

Finally, underlying beliefs relating to the criminal justice system and, more specifically, to the reliability of evidence and trustworthiness of testimony may influence assessments of memories of others. For example, people who have high confidence that the justice system tends to lead to correct outcomes may have an inherent trust in memory in the eyewitness context and therefore may have a tendency to classify identifications as correct, leading to higher accuracy in assessments of correct identifications but lower accuracy in assessments of false identifications.

In this work, we examine how each of these four sets of characteristics and beliefs are associated with accuracy in assessments of identifications. We also conduct a linguistic analysis to examine the reasoning underlying memory assessments and the extent to which different types of reasoning (relating to these characteristics and beliefs and more generally) are associated with accurate or inaccurate assessments.

### 1.4. The Current Study

This study is relatively exploratory and is intended to build on existing work to begin to provide information that can be drawn on in seeking to better understand assessments of identifications made by others and to develop intervention to improve these assessments in the legal context. Participants in the study (members of the public in the USA) examined “real” correct and false identifications made by witnesses to a mock crime, and their assessments were examined to provide new insight in relation to three key research questions.

First, how effective are lay assessors at distinguishing between accurate and inaccurate identification evidence? To answer this question, we examine overall levels of accuracy in assessments of identifications, and also the extent to which participants’ confidence in their assessments of identifications is related to the accuracy of those assessments (something with the potential to be important in jury deliberations).

Second, can instructions utilizing anchors informed by psycho-legal research improve the way that assessors utilize confidence as a cue in assessing identification evidence and ultimately improve the accuracy of assessments? To answer this question, we compare the associations between witness confidence and judgments of identification accuracy in participants who have received no instruction, a basic instruction (warning jurors that confident identifications may not be accurate), and an anchor instruction (utilizing benchmarks outlined in Wixted & Wells, 2017 relating to the relationship between confidence and accuracy generally in witness identifications).

Third, which characteristics and beliefs of assessors facilitate more (or less) accurate assessments of identification evidence? To answer this question, we examine associations between characteristics and beliefs and the accuracy of assessments of identifications, and conduct a linguistic analysis to examine reasoning associated with higher and lower accuracy in assessments.

## 2. Materials and Methods

### 2.1. Design

Each participant viewed and made assessments in relation to four identifications (and accompanying testimony), selected from a pool of identifications from a mock crime study in which “witnesses” saw a staged video of a person stealing a laptop and, approximately five minutes later, sought to identify the person who they had seen take the laptop from a six-person lineup with a not present option (see below for more information in relation to the underlying mock crime study). The specific identifications that each participant saw were randomly selected from groups in the larger pool of identifications, such that the four identifications seen by each participant included a randomly selected true identification and a false identification from each of two stimulus sets (although participants were not given any information in relation to the number of true and false identifications that they would see). The instructions that participants saw prior to viewing the identifications varied between subjects. Participants either saw no instructions, saw a traditional instruction in which they received warnings about potential weaknesses in identification evidence, or saw an ‘anchor’ instruction in which they received information about the proportion of witnesses who tend to be accurate at different levels of confidence.

The traditional instruction stated: “*This information is designed to assist you in making your assessments. Please read this information very carefully and consider it in evaluating the identifications. A witness who is honest and convinced in his or her own mind may be wrong. A witness who is convincing may be wrong. A witness who is able to recognise the defendant, even when the witness knows the defendant very well, may be wrong.*” The anchor instruction stated: “*This information is designed to assist you in making your assessments. Please read this information very carefully and consider it in evaluating the identifications. Generally, under fair conditions, eyewitness confidence is predictive of accuracy. On average: when eyewitnesses have low confidence that their identification is correct, identifications turn out to be incorrect around 35% of the time (and correct around 65% of the time)—when eyewitnesses have moderate confidence that their identification is correct, identifications turn out to be incorrect around 25% of the time (and correct around 75% of the time)—when eyewitnesses have high confidence that their identification is correct, identifications turn out to be incorrect around 5% of the time (and correct around 95% of the time). Note that these are average figures, rather than figures that apply to the specific identifications in this study. They are intended to provide context for you to consider in assessing the likelihood that an identification is accurate in this particular case where the probability of a particular identification being inaccurate may be higher or lower than average.*” 

### 2.2. Participants

Participants were 498 adults recruited from the Prolific survey platform, who received GBP 6.80 (approximately USD 9) for completing the experiment. We had initially sought to recruit 500 participants so that each identification in our study would be viewed by at least 50 participants, but under-recruited very slightly since some participants on Prolific did not provide complete responses.

To be eligible for the survey, participants were required to live in the United States, to have a Prolific approval rating of at least 90%, to have completed at least 10 previous Prolific submissions, and could not have participated in the underlying mock crime work. Two participants were excluded for failing two or more of the three attention check questions in the survey. Nine participants were excluded due to verbal responses that were either nonsensical, repetitive (e.g., giving exactly the same answer multiple times), or identical to responses provided by other participants. The final sample therefore consisted of 487 participants (229 male, 251 female, 7 other gender/prefer not to say, 331 white ethnicity, 122 black/African/Caribbean ethnicity, 11 Asian ethnicity, 22 mixed/other ethnicity/prefer not to say, M_age_ = 38.73, SD = 12.26, range = 18–83). The Faculty of Humanities and Social Sciences Research Ethics Committee at the University of Exeter approved this research.

### 2.3. Materials and Procedure

**Identifications: Stimuli Generation.** Identifications shown to individual participants were selected from a set of 40 identifications, including 10 true and 10 false identifications from each of two stimulus sets (stimulus set A, involving a white target, and stimulus set B, involving a black target). Each set of 10 identifications were randomly selected from the corresponding categories in a larger set of 110 identification decisions from a mock crime study (the overall set of identification decisions from that set was larger [N = 485] and also included non-identifications and incorrect identifications from a target present lineup, both of which were excluded as potential stimuli for the purposes of this study). In the mock crime study, witnesses viewed a video lasting approximately two minutes in which a target stole a laptop while the owner of the laptop stepped out of the room to answer a phone call. The target in the video was clearly visible for approximately one minute. After a five-minute buffer task, they were then asked to identify the person that they had seen take the laptop from a six-person lineup including a “not present” option. They then answered a series of questions in relation to their identification (or non-identification), including: “How confident are you in your identification?” “What led to your decision?” “How well did you see the person who took the laptop?” “How difficult was it for you to figure out which person in the lineup was the person who took the laptop?” “How much attention were you paying to the face of the person who took the laptop?” And “How good is your memory generally for the faces of strangers?” (broadly mirroring the information requested in Wells & Bradfield, 1998). Participants were instructed to record themselves (on video) while making the initial identification and while answering each of these questions. In the overall data for stimulus set A, there were 27 false identifications from target absent lineups and 31 correct identifications. In the overall data for stimulus set B, there were 16 false identifications from target absent lineups and 36 correct identifications. Participants were also asked to provide their confidence in their identification on an 11-point written scale from 0 (not at all confident) to 100 (very confident), increasing in increments of 10. Levels of confidence in the overall set of 110 identifications from the mock crime work, and the sub-set of 40 identifications used in this study, are outlined in Table 1.

**Identifications and Assessment Questions.** Each participant in this study first received information about the event that the witness in the mock crime study observed. Specifically, they were told that, in a previous experiment, participants (“witnesses”) watched a video in which they saw a person taking a laptop while its owner was out of the room. They were informed that the video was approximately two minutes long, that the person who took the laptop was visible for about a minute, that approximately five minutes after viewing the video, the witness was asked to make an identification of the person who they saw, and that the identification was made from a lineup containing six photographs of faces and with a not present option. They were then told that they would see four identifications made by participants who saw different videos, and therefore different people taking the laptop, and that they would be asked to assess the likely accuracy of each identification.

Participants then viewed each of the four identification and testimony sets allocated to them in a randomized order. After viewing each identification, they answered four questions in relation to it. First, they were asked to indicate how likely they thought it was that the person the witness identified was the person who took the laptop in the video they saw on an 11-point scale from 0 (not at all likely) to 100 (very likely), in increments of 10. Next, they were asked to provide a dichotomous judgment of whether the identification they saw was correct or incorrect, and to indicate how confident they were in that judgment on an 11-point scale from 0 (not at all confident) to 100 (very confident), in increments of 10. Finally, they were asked to explain in as much detail as they could why they made their assessment of the witness identification. After they had watched all four video sets and answered all four sets of assessment questions, they moved on to complete the individual difference measures.

### 2.4. Individual Difference Measures

Participants then completed several individual difference measures in a set order.

First, they completed the Eyewitness Metamemory Scale, a 23-item questionnaire made up of three scales measuring peoples’ perceptions of their own memory. These three scales measure memory contentment (satisfaction with their ability to remember faces; α = 0.84), memory discontentment (doubt in their ability to memory faces; α = 0.89), and memory strategies (their use of strategies to remember faces; α = 0.69) (Saraiva et al., 2019). Second, they completed the Autism Quotient, a 50-item scale developed to assess the presence of autism spectrum traits in adults (Baron-Cohen et al., 2001; α = 0.70), with five subscales—communication (α = 0.64), social (α = 0.72), imagination (α = 0.52), details (α = 0.65), and attention switching (α = 0.40). This measure was included not with a view to diagnosing autism or to assessing autism, but as an indirect measure of gist (meaning focused) vs. verbatim (detail focused) cognitive processing (see discussion above, and Reyna & Brainerd, 2011). Next, they completed a memory questionnaire, examining their beliefs about the factors that do (and do not) affect memory performance (Benton et al., 2006). The questionnaire consists of 30 items, with three response options (“Generally True,” “Generally False,” and “I don’t know”). Finally, they completed the Pretrial Juror Attitudes Questionnaire (PJAQ; α = 0.88), a 29-item scale developed to measure individual differences in attitudes relevant to juror decision making, with six subscales: confidence in the criminal justice system (α = 0.76), conviction proneness (α = 0.73), cynicism to the defense (α = 0.74), racial bias (α = 0.42), social justice attitudes (α = 0.28), and belief in innate criminality (α = 0.65) (Lecci & Myers, 2008).

## 3. Results

In our analyses we examined overall accuracy and, in addition, accuracy in assessments of true identifications and false identifications separately. This approach was taken since factors associated with assessments of identifications may have a differential impact on the accuracy of assessments of true and false identifications (e.g., confidence in the accuracy of eyewitness memory may lead to greater accuracy in the assessment of true identifications but lower accuracy in the assessment of false identifications).

### 3.1. Overall Accuracy (And the Impact of Instructions on Overall Accuracy)

Overall, four participants (0.8%) were incorrect in all of their assessments (i.e., characterized the correct identification decisions that they saw as false identifications, and the false identifications that they saw as correct identifications), 37 participants (7.6%) were correct in one assessment and incorrect in three assessments, 137 participants (28.1%) were correct in two assessments and incorrect in two assessments, 182 participants (37.4%) were correct in three assessments and incorrect in one assessment, and 127 participants (26.1%) were correct in all of their assessments. In stimulus set A, higher witness confidence was predictive of lower accuracy in the assessment of correct identifications (B = −0.027, SE = 0.013, Wald = 4.508, *p* = 0.034, OR = 0.973, 95% CI [0.949, 0.998]) and in the assessment of false identifications (B = 0.032, SE = 0.005, Wald = 35.89, *p* < 0.001, OR = 0.969, 95% CI [0.958, 0.979]). In stimulus set B, higher witness confidence was predictive of greater accuracy in the assessment of correct identifications (B = 0.051, SE = 0.005, Wald = 94.33, *p* < 0.001, OR = 1.052, 95% CI [1.041, 1.063]) but higher witness confidence was not predictive of accuracy in the assessment of false identifications (B = −0.002, SE = 0.005, Wald = 0.155, *p* = 0.694, OR = 0.998, 95% CI [0.989, 1.007]).

**Figure 1:**
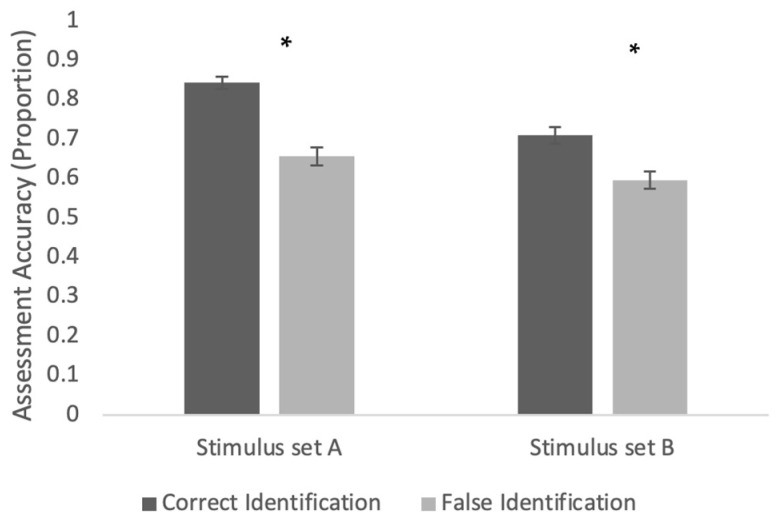
Interaction between identification accuracy and stimulus set in predicting assessment accuracy. * *p* < 0.05.

In order to further probe accuracy and the conditions conducive to more accurate judgment, a generalized estimating equation model with an independent correlation structure was used in which the instructions (no instructions, basic instructions, anchor instructions), stimulus set (stimulus set A, stimulus set B), and underlying accuracy (correct identification, false identification) were used to predict the accuracy of assessor judgments. This analysis revealed a number of significant main effects and interactions. First (and consistent with prior work, e.g., Lindsay et al., 2000), there was a significant main effect of identification accuracy (Wald χ^2^(1) = 52.86, *p* < 0.001), such that participants were significantly more likely to be accurate in their assessments of correct identifications than in their assessments of false identifications (M_true_ = 0.78, SE = 0.02; M_false_ = 0.63, SE = 0.01). Second, there was a significant main effect of stimulus set (Wald χ^2^(1) = 25.91, *p* < 0.001), such that assessments of identifications from stimulus set A were more accurate than assessments of identifications from stimulus set B (M_setA_ = 0.75, SE = 0.01; M_setB_ = 0.65, SE = 0.02). These effects were moderated by a significant interaction between identification accuracy and stimulus set (Wald χ^2^(1) = 7.03, *p* = 0.008). Identification accuracy showed the same pattern over both stimulus sets (with assessments of correct identifications more accurate than assessments of false identifications), but this pattern was more pronounced in stimulus set A (see Figure 1).

**Figure 2:**
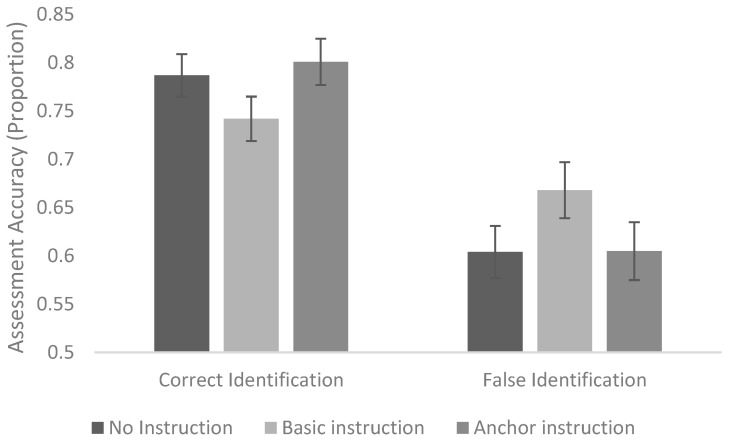
Interaction between identification accuracy and instruction condition in predicting assessment accuracy.

Finally, there was a significant interaction between instruction condition and identification accuracy (Wald χ^2^(2) = 6.73, *p* = 0.035; Figure 2). Differences in assessment accuracy were not significant between instruction conditions for correct identifications or for false identifications. However, a visual inspection of the data suggests that the basic instruction decreased accuracy in assessments of correct identifications and increased accuracy in assessments of false identifications when compared to no instruction, while the anchor instruction increased accuracy in assessments of correct identifications and did not change accuracy in assessments of false identifications.

Finally, we used a series of logistic regression models to examine whether participants’ own confidence in the accuracy of their identification assessments significantly predicted the actual accuracy of those assessments. In both stimulus sets participant confidence in their assessments was only predictive of accuracy (such that people were more confident in accurate assessments than incorrect assessments) for assessments relating to correct identifications (stimulus set A false identifications B = 0.002, SE = 0.005, Wald = 0.131, *p* = 0.718, OR = 1.002, 95% CI [0.993, 1.011]; stimulus set B false identifications B = 0.004, SE = 0.004, Wald = 0.996, *p* = 0.318, OR = 1.004, 95% CI [0.996, 1.013]; stimulus set A correct identifications B = 0.049, SE = 0.007, Wald = 48.73, *p* < 0.001, OR = 1.050, 95% CI [1.036, 1.065]; stimulus set B correct identifications B = 0.051, SE = 0.006, Wald = 76.84, *p* < 0.001, OR = 1.053, 95% CI [1.041, 1.065]).

### 3.2. Witness Confidence and the Relationship Between Instructions and Witness Confidence

We examined the overall relationship between witness confidence and assessment accuracy using a series of four logistic regression models. The results were inconsistent between our stimulus sets, and, in some instances, not in line with expectations. In stimulus set A, higher witness confidence was predictive of lower accuracy in the assessment of correct identifications (B = −0.027, SE = 0.013, Wald = 4.508, *p* = 0.034, OR = 0.973, 95% CI [0.949, 0.998]) and in the assessment of false identifications (B = 0.032, SE = 0.005, Wald = 35.89, *p* < 0.001, OR = 0.969, 95% CI [0.958, 0.979]). In stimulus set B, higher witness confidence was predictive of greater accuracy in the assessment of correct identifications (B = 0.051, SE = 0.005, Wald = 94.33, *p* < 0.001, OR = 1.052, 95% CI [1.041, 1.063]) but higher witness confidence was not predictive of accuracy in the assessment of false identifications (B = −0.002, SE = 0.005, Wald = 0.155, *p* = 0.694, OR = 0.998, 95% CI [0.989, 1.007]).

We also conducted the same regressions (reported in the same order), including instruction condition and the interaction between instruction condition and witness confidence as predictors of accuracy in each of the four logistic regressions. The interaction term was not significant in any of these regressions (*p* = 0.496; *p* = 0.742, *p* = 0.927; *p* = 0.337, respectively), indicating that instructions did not alter the relationship between confidence and accuracy in participants assessments.

### 3.3. Associations Between Assessor Characteristics and Beliefs and Accuracy

Assessor experiences with memory were not significantly associated with assessment accuracy; however, we found significant relationships between our other measures of assessor characteristics and beliefs and assessment accuracy, as detailed below.

#### 3.3.1. Beliefs About Memory

To assess associations between memory beliefs and accuracy assessments, we coded percentage agreement with memory statements, combining “generally false” and “don’t know” responses in one category (as in Benton et al., 2006). The percentage of our participants who agreed with each statement is provided in Table 2, alongside equivalent percentages for the experts surveyed by Kassin et al. (2001) (and utilized as a comparison group in Benton et al., 2006). The table also indicates where endorsement of a particular statement as being “generally true” was associated with higher accuracy in assessments of memory accuracy in our data.

#### 3.3.2. Gist vs. Verbatim Processing (Autistic Traits)

Overall assessment accuracy significantly correlated with autistic traits, such that those who showed higher levels of autistic traits tended to make less accurate assessments (*r*(487) = −0.10, *p* = 0.041; although correlations between accuracy and AQ subscales were not significant, this correlation appeared to be driven largely by items in the attention to detail and imagination subscales since correlations between these subscales and accuracy approached significance [*p* = 0.072, and *p* = 0.078, respectively]). Accuracy in classifying false identifications as false correlated with the AQ imagination subscale (such that those with limitations in imagination were less likely to correctly classify false identifications as false; *r* [487] = −0.11, *p =* 0.02). Accuracy in classifying true identifications as true did not correlate significantly with the overall AQ score, or scores on any AQ subscales.

#### 3.3.3. Beliefs About the Criminal Justice System

The overall assessment accuracy significantly correlated with beliefs in innate criminality, such that those who had stronger beliefs in innate criminality tended to make less accurate assessments (*r*(487) = −0.10, *p* = 0.027). Accuracy in classifying false identifications as false correlated with the overall PJAQ score (such that those with higher scores on the PJAQ were less likely to correctly classify false identifications as false; *r* [487] = −0.14, *p =* 0.002), as well as with scores on the PJAQ system confidence, conviction proneness, racial bias, and innate criminality subscales (all in the same direction as the overall scale correlation; *r* [487] = −0.12, *p* = 0.007, *r* [487] = −0.11, *p* = 0.013, *r* [487] = −0.13, *p* = 0.005 and *r* [487] = −0.15, *p* = 0.001, respectively). Accuracy in classifying true identifications as true correlated with PJAQ system confidence and PJAQ conviction proneness, such that higher scores on each of these subscales was associated with greater accuracy in classifying true identifications as true (*r* [487] = −0.112, *p* = 0.014 and *r* [487] = −0.09, *p* = 0.049, respectively).

#### 3.3.4. Reasoning Underlying Assessments

To examine reasoning associated with higher and lower accuracy in assessments, we conducted a corpus linguistic analysis of the participants’ responses to the question: “Please explain in as much detail as you can why you made this assessment of the witness identification.” We compiled a corpus with those responses (corpus ACBC). In a further analysis, we divided the responses into two groups based on the overall accuracy of their four assessments: the above-chance group (AC; n = 309), with participants scoring three (n = 182) and four (n = 127), and the chance + below-chance group (CBC; n = 178), with participants scoring zero (n = 4), one (n = 37), or two (n = 137).

We used the corpus software *AntConc* (Anthony, 2024) to conduct a keyword analysis to identify key topics and features in the different corpora. A keyword is defined as “a word that occurs statistically more frequently in one file or corpus when compared against another comparable or reference corpus” (Baker, 2010, p. 104). The reference corpus is Corpus AmE06, a one-million-word reference corpus of general written American English built in 2011 with most of its texts publicized in 2006 (Potts & Baker, 2012). The keywords meet the threshold of *p* < 0.05 and were sorted by likelihood, which is measured by log-likelihood (4-term).

We first conducted a keyword analysis by comparing corpora ACBC and AmE06, examining the top 100 keywords (Table A1 in Appendix A) to identify the main themes in participants’ reasoning, regardless of their overall scores. The keywords indicate that the reasoning centers around witness identification related to someone who stole a laptop. Several factors are important in their assessment of witness identification, such as confidence (confident, sure, confidence, unsure, certain), memory (memory, good, strangers), attention (paying, paid, attention), view (camera, face, faces), and difficulty in identification (difficult).

Further analysis focused on the comparison between the corpora AC and CBC. Differences have been identified in their top 100 keywords: they share 80 words but differ in 20 (Table A2). All 80 shared words are included in Table A1.

As the keyword list was generated based on keyness (the degree to which each appeared important), calculated using log-likelihood tests, it includes words that are not frequently used in the two corpora. We therefore also examined word lists, which are generated based on frequency. The comparison of the top 100 words in AC and CBC reveals that they share 89 words, with each having 11 words that are not shared (Table 3). The shared words include some listed in Table A1, as well as many functional words like “the”, “of”, “in”, etc.

**Figure 3:**
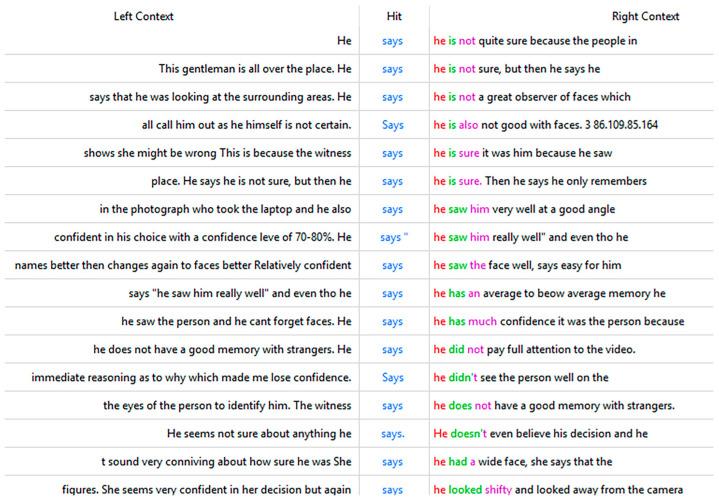
The context of “says” in corpus AC.

Four words that were identified as important in AC responses (but not CBC responses), which provide insight into the information that participants who performed above chance relied on are “says,” “difficult,” “really,” and “strangers” (the last three of these words are also in Table A2). An analysis of how these words were used provides further insight into participant reasoning. An analysis of the words frequently used alongside “says” shows that AC participants were frequently referring to what witnesses said about their view, difficulty, confidence, attention, and memory (Table 4), which can also be seen more broadly in the context in which “says” was used (Figure 3). We found similar results for the word “really”.

**Figure 4:**
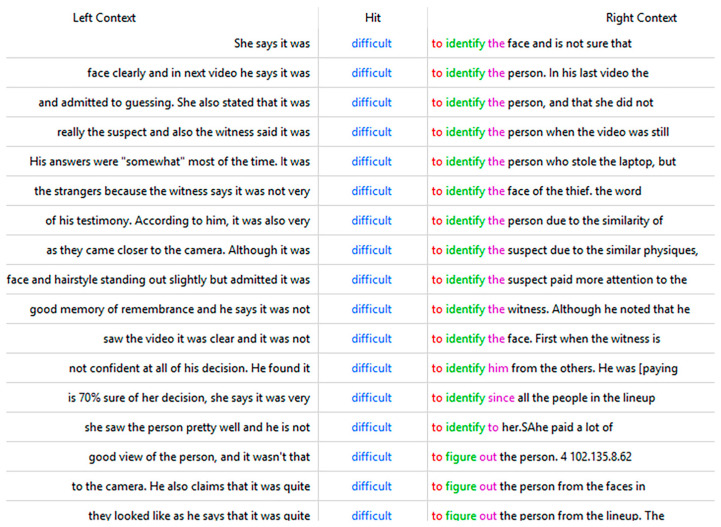
The context of “difficult” in corpus AC.

AC participants’ attention to difficulty and the witness’s memory experience are also reflected in the keyness and frequency of “difficult” and “stranger,” which appear in both Table 3 and Table A2. An examination of the context of “difficult” reveals that it is mainly used to refer to the witness’s statement about the difficulty level that they experienced in identification (Figure 4). AC participants deem this an important standard in witness evaluation. For example, one AC participant stated: “*She says it was difficult to identify the face and is not sure that the face is in the lineup. She also says that she does not have a good facial memory.*”

**Figure 5:**
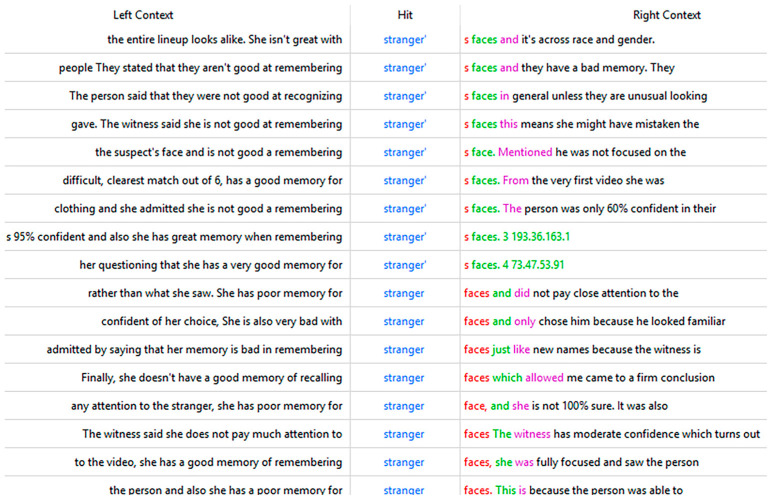
The context of “stranger” in corpus AC.

The word “stranger” occurs frequently in corpus AC because it is related to witnesses’ statements about their memory of strangers’ faces (Figure 5), which clearly shows that they paid attention to and utilized the responses in relation to the general ability to remember the faces of strangers.

**Figure 6:**
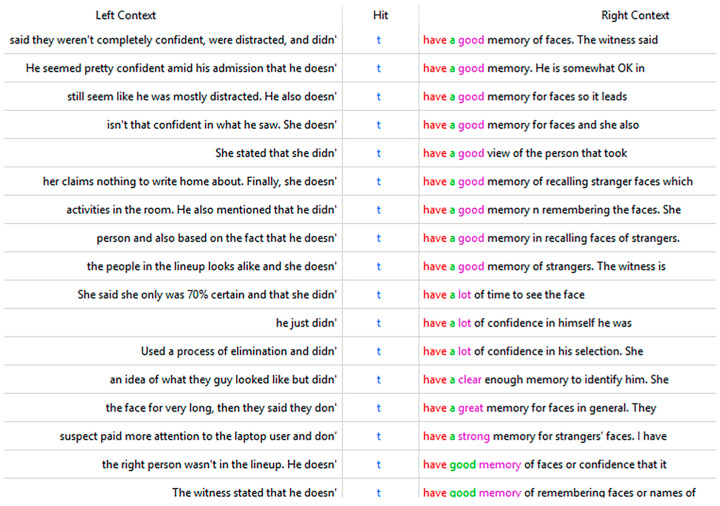
The context of “t” in corpus AC.

AC participants not only pay attention to what the witness “says” in response to those questions but also impose high standards when evaluating these responses to decide whether to accept their identification. This is demonstrated by the keyness and/or frequency of negation marker “t,” (Table A2) “only,” (Table 3) and “elimination” (Table A2). For AC responses, the most salient aspect shared by Table 3 and Table A2 is the negation markers, including “t,” and “didn” in Table A2 and “wasn” in Table 3. An examination of the collocates and context of “t” (Table 5 and Figure 6) shows that the main aspects of testimony being negatively evaluated include confidence, attention, and memory.

”Only” also indicates AC participants’ high standards in evaluation. Its context reveals the participants’ dissatisfaction with the witnesses’ confidence levels and limited visual information. One representative response is “*The witness was only 80% sure this was the criminal that took the laptop. I personally need a 100% sure from witness to really believe the suspect is the criminal.*”. Similarly, another participant responds, “*This person is only 85% confident and basically judging one from his or her facial appearance is not making sense at all*”. Another word that indicates AC participants’ high and rigorous standards is “elimination” (18 occurrences), which, as an identification method, is criticized by AC participants. This indicates attention to the process of the identification. For example, “*She used a process of ‘elimination’ rather than a process of ‘identification’ and was unsure of herself. She lacked confidence in her answer and did not seem reliable*”.

Unlike AC participants’ attention to what witnesses said about confidence, memory, difficulty, view, and attention levels, CBC participants seemed to focus on “details,” primarily in the witness’s description of the subject. This can be seen in the frequency of “details” (Table 3). When its singular form is also investigated, we find that the lemma’s combined occurrences amount to 79 times in total in corpus CBC. This frequency would rank it among the top 55 words on the word list, which includes all functional words. This highlights the importance of detail in CBC participants’ evaluations.

The collocates (shown in Table 6) show that “details” mainly occurs in contexts where the witnesses provided details about the suspect. Such actions are deemed important by CBC participants, making their evaluation of the witness lean towards the positive. An exemplary response is “*The witness appeared consistent and specific in their reasoning. They referred to clear details about the suspect’s facial structure and features that matched the defendant in the lineup.*” On the contrary, when a witness fails to provide details, participants tend to give a negative evaluation. For example, “*However, some aspects warrant scrutiny. The witness’s basis for identification—resemblance to the person who took the laptop—is somewhat vague, lacking specific details about distinguishing features.*” The keyness of “explanation”, “shape”, “match(ed)”, and “specific” (Table A2) and the frequency of “able” (Table 3) also point to CBC participants’ attention to details as shown in the following two examples.

*“The witness gave a detailed and clear explanation for their identification, mentioning specific features like the distinct red jacket and the way the person moved. These specific details suggest that the witness paid close attention to the suspect’s characteristics, which supports the likelihood of accuracy.”*

*“…he was able to grab the shape of his head and a view detail even though he is not 100 percent confident, but he was able to identify number 5 to be the culprit. that tells a lot”*

The above examples regarding attention to details also show CBC participants’ tendency to make inferences, even though clear statements have been explicitly given by the witnesses about their confidence and attention levels. For these CBC participants, the witnesses’ own descriptions of their levels of confidence and attention are not as important as the participants’ inferences based on their observations of the witnesses’ verbal and non-verbal cues. This is further supported by the keyness of “hesitation” in the CBC corpus (Table A2). The following two are examples of how they evaluate “hesitation”.

*“I think he seems somewhat uncertain but he does say he has a generally good memory for faces of people he meets. I think it’s quite possible he picked the right one but there is also a possibility that he was wrong, His hesitation made me wonder about how sure he really was.”*

*“I believe the witness made the correct identification because she appeared confident in her decision. She showed no signs of hesitation at all. Given the clarity and the speed of her choice, along with the absence of any cues or influence, I feel confident that their identification was accurate.”*

Consistency is also an important factor in CBC participants’ evaluations, as “consistent” is a keyword that stands out in Table A2. This suggests that, unlike AC participants, who focus on an identification itself, CBC participants also attach importance to the witness’s credibility or, more specifically, honesty, which again involves inferences. For example, the following are two responses from a participant with an overall score of one:

*“He seemed consistent, but admitted to missing key features and being unable to say with confidence which was the person who stole the laptop. Admitted to not being good with faces as well! When he said he was 60% confident in addition to not being good with faces, I had my mind made up to not fully rely on his testimony! He was honest, which is vital!”*

*“Inconsistent markers in the first few sets of answers. He claimed he seen a direct shot of the person who stole the laptop, then after that had said he looked similar to the picture he chose from. That choice should have been more direct, instead of seeming more like a choice or opinion. Regardless of the circumstance! It should have been more direct! Based on the disorganized thought process/speaking (mixing up words & numbers slightly) I would also say this is a little bit of a red flag, since we are seeking honesty here. I personally find these two things inconsistent when determining if someone is correct or incorrect in what they say.”*

## 4. Discussion

### 4.1. How Effective Are Lay Assessors at Distinguishing Between Accurate and Inaccurate Identification Evidence?

Results support prior research (e.g., Lindsay et al., 2000) by demonstrating a general tendency to over-estimate the reliability of identification evidence. While the majority of our sample performed better than chance in their four assessments, there remained a clear risk of inaccuracy in assessments, particularly in the assessment of false identifications, where participants believed that false identifications were correct 37% of the time. Importantly, our results also showed that participants’ own confidence in the accuracy of their assessments of false identifications was not predictive of the actual accuracy of those assessments. This reality has the potential to be problematic in the jury context, where more confident jurors are likely to be more persuasive than less confident jurors (despite our results suggesting that they are not more likely than less confident jurors to be correct) (on the role of confidence in jury deliberations, see Devine, 2012; Helm, 2024). There is therefore a risk that in some cases jurors who accurately classify a memory as false are persuaded that the memory is true by jurors who have inaccurately classified the memory as true. Future work should further probe our metacognitive ability in evaluating the accuracy of our assessments relating to others.

Another interesting finding from this study in relation to accuracy was that assessments of witness accuracy were more accurate in cases relating to one stimulus set than in cases relating to another stimulus set. This finding suggests that there may be specific categories of case in which assessors are particularly prone to mistakes in the assessments of identification evidence. It may be important that the identifications that participants performed more poorly at assessing were identifications of ethnically black targets. It is possible that participants overall had less experience of memorability and cues associated with memorability in relation to those targets and that this led to poorer performance as assessors of memory evidence. This study was not set up to test predictions relating to race and this explanation is only one of many possible explanations; however, prior work has found that ethnically black assessors perform better in assessing identifications of black targets than ethnically white assessors (Helm & Spearing, 2025). The relationship between target race and overall accuracy of juror evaluations of witness memory should be examined further in future work.

### 4.2. Can Instructions Utilizing Anchors Informed by Psycho-Legal Research Improve the Way That Assessors Utilize Confidence as a Cue in Assessing Identification Evidence?

This study did not find any evidence to support the effectiveness of our instructions to improve the utilization of confidence as a cue in assessing identification evidence or to improve the accuracy of assessments, although we did find limited evidence that traditional instructions may increase skepticism in assessments (essentially resulting in slightly more accurate assessments of false identifications and slightly less accurate assessments of correct identifications). One possible reason that instructions were ineffective may be the number of cues other than confidence that participants relied on in making their assessments of identifications (identified in our linguistic analysis), meaning that, even if their views on how to utilize confidence had been effectively changed by the instructions, this would not have had a significant overall impact on assessments. Another reason that instructions could have been ineffective is that they were relatively brief and not sufficiently detailed to alter participant intuitions relating to confidence in a meaningful way that they could apply in context (e.g., Helm, 2021a). It is possible that more detailed “training-based” instructions utilizing a similar approach may be more effective. Moving forward, the design of new instructions may also be informed by the results of this work, which provide new insight into characteristics and reasoning associated with assessment accuracy (as discussed below).

### 4.3. Which Characteristics and Beliefs of Assessors Facilitate More Accurate Assessments of Identification Evidence?

This study provides some important early insight into what might make assessors (including jurors) better or worse at assessing identification evidence in the legal context. The relationship between autistic traits (used in our study as a proxy for more verbatim-based reasoning) and accuracy, specifically that higher scores on the AQ were associated with lower overall accuracy, supports the suggestion that there may be certain cognitive differences that make us better or worse at making assessments of the likely accuracy of the memory of others (rather than just biasing judgments in a particular direction). A possible explanation for the relationship, linked to our predictions relating to reliance on gist, is that those with more verbatim processing (indicated here by higher autistic traits) focus more on details and less on a meaningful evaluation of the situation of the witness. This explanation is somewhat supported by the subscales of the AQ that seem to be driving the identified relationship (attention to detail and lack of imagination), and, also, the finding from our linguistic analysis that focus on detail in this case appears to be related to lower levels of accuracy. However, further research should examine this relationship in order to understand it further and to test these potential explanations. The fact that beliefs in innate criminality were related to accuracy, such that greater beliefs in innate criminality were associated with lower overall accuracy, may be driven by decisions being influenced by bias (specifically towards classifying identifications as true) rather than more precise assessment. However, an interesting note in this regard is that beliefs in innate criminality were not significantly associated with increased accuracy in the classification of true identifications.

Endorsement of a number of beliefs (all of which are generally endorsed by experts), specifically “eyewitnesses sometimes identify as a culprit someone they have seen in another situation or context,” “An eyewitness’s confidence can be influenced by factors that are unrelated to identification accuracy,” and “Young children are more vulnerable than adults to interviewer suggestion, peer pressures and other social influences,” were also associated with greater accuracy. These statements are all related to a relatively cautious and nuanced approach towards the evaluation of memory, recognizing ways in which memory can be flawed. The greater accuracy may therefore be a result of a broad cautious and nuanced approach rather than the result of specific endorsement of statements themselves. However, further research should examine whether these beliefs in particular are predictive of accuracy and, if they are, could build on this insight in designing instructions to educate the jury in a way that improves the accuracy of assessments.

Results also provide new and important insight into the reasoning underlying more accurate and less accurate assessments of identification evidence. Overall, results suggest that a focus on what witnesses say, particularly in relation to factors that are likely to be probative, including self-reported confidence, memory experience, view, and difficulty level, has the potential to increase accuracy while a reliance on inferences from how a witness explains their identification, including the detail that they provide about the suspect or non-verbal cues, has the potential to decrease accuracy. In relation to non-verbal cues, these findings are consistent with research in the literature on deception detection, which shows that the non-verbal cues that people tend to pick up on in making assessments in relation to the honesty of others have little to no probative value and that reliance on them can decrease the quality of assessments (see, for e.g., DePaulo et al., 2003; Vrij et al., 2019; Mann et al., 2004 [in the context of police assessments]; McKimmie et al., 2014).

### 4.4. Limitations, Future Directions, and Policy Implications

As noted in the introduction, this study is relatively exploratory and is intended to build on existing work to begin to provide information that can be drawn on in seeking to better understand assessments of identifications made by others and to develop an intervention to improve these assessments in the legal context. The results should be interpreted with the exploratory nature of this study in mind and should be viewed as early evidence providing insight that can be built on in future work to inform policy rather than as conclusive findings that will generalize more broadly. The study also has several specific limitations in relation to generalizability to the legal context in practice. First, all participants in our study were viewing identifications made in relation to the same mock crime. Although we used two stimulus sets (one of which included an ethnically white target and one of which included an ethnically black target) and 40 individual identifications, all witnesses viewed a video of a target stealing a laptop in which the target was clearly visible for approximately one minute. Identifications following viewing this crime may have particular characteristics that make them different from identifications in different contexts (including events viewed in person rather than on camera, although see Pozzulo et al., 2008; Rubínová et al., 2021), and future work should examine the extent to which results generalize to assessments of identifications made after viewing other crimes (including crimes involving factors such as violence, with the potential to influence memory; see, for e.g., Bogliacino et al., 2017). In addition, in this study participants viewed witnesses providing information in response to questions about their identification in a neutral context, and in video form. In real adversarial trials evidence would be provided through direct and cross examination, and this form of eliciting information could change the cues that assessors rely on and, relatedly, alter the accuracy of their assessments. In addition, witnesses would typically appear in person, meaning that assessors may pick up on different cues, particularly in the case of non-verbal cues, which may appear differently in person.

Despite these limitations and the need for further research to (1) replicate findings, (2) generalize findings to new contexts, and (3) examine findings in more ecologically valid contexts, this study provides important and novel insight about the general ability of non-experts to evaluate the memory of others that has the potential to be important not only in legal contexts but in any other contexts in which we make judgments about the memory of others (e.g., in education). In the legal context, the findings, when extended in future work, have the potential to inform more effective practice and procedure surrounding the evaluation of memory evidence, including to inform the design of effective instructions to improve the abilities of jurors in this role. For example, the work suggests that it may be helpful to instruct jurors to rely on what eyewitnesses say rather than to make inferences from their verbal and non-verbal behavior and that, if such instructions are ineffective, providing evidence in written form rather than verbal form may be helpful to consider. The findings may also be applied in the context of voir dire in cases in which eyewitness identification testimony is particularly important. For now, the findings add to an existing body of work that demonstrates that humans are not *inherently* effective evaluators of the memories of others, and that legal systems should not rely on our ability to assess the likely accuracy of the memory of others in making important legal determinations.

## Figures and Tables

**Table 1:** Descriptive statistics for identifications.

Title 1	All Identifications		Stimuli Identifications	
	Set A	Set B	Set A	Set B
Confidence in Correct Identifications (SD)	85.81 (15.87)	76.11 (22.59)	84.00 (10.75)	77.00 (25.84)
Confidence in Incorrect Identifications (SD)	64.81 (18.05)	56.88 (25.75)	58.00 (20.44)	53.00 (22.14)

**Table 2:** Agreement rates of participants compared to agreement rates of experts from Kassin et al. (2001), and relationship between endorsement and accuracy.

Statement	% Participants (n = 487)	% of Experts (Kassin et al., 2001; n = 64)	Endorsement of Statement Significantly Associated with Higher Accuracy in Identification Assessments
Very high levels of stress impair the accuracy of eyewitness testimony.	81.5	60	No
The presence of a weapon impairs an eyewitness’s ability to accurately identify the perpetrator’s face	62.2	87	No
The use of a one-person show-up instead of a full lineup increases the risk of misidentification.	59.1	74	No
The more members of a lineup resemble the suspect, the higher is the likelihood that identification of the suspect is accurate.	42.7	70	No
Police instructions can affect an eyewitness’s willingness to make an identification.	80.3	98	No
The less time an eyewitness has to observe an event, the less well he or she will remember it.	65.1	81	No
The rate of memory loss for an event is greatest right after the event and then levels off over time.	50.7	83	No
An eyewitness’s confidence is not a good predictor of his or her identification accuracy.	50.7	87	No
Eyewitness testimony about an event often reflects not only what they actually saw but also information they obtained later on.	75.8	94	No
Judgements of color made under monochromatic light (e.g., an orange street light) are highly unreliable.	51.7	63	No
An eyewitness’s testimony about an event can be affected by how the questions put to that witness are worded.	81.7	98	No
Eyewitnesses sometimes identify as a culprit someone they have seen in another situation or context.	71.3	81	Yes (M_true_ = 2.86, SD = 0.93, M_other_ = 2.66, SD = 0.95, f(486) = 4.763, *p* = 0.030)
Police officers and other trained observers are no more accurate as eyewitnesses than is the average person.	49.9	39	No
Hypnosis increases the accuracy of an eyewitness’s reported memory.	41.3	45	No
Hypnosis increases suggestibility to leading and misleading questions.	54.6	91	No
An eyewitness’s perception and memory for an event may be affected by his or her attitudes and expectations.	79.5	92	No
Eyewitnesses have more difficulty remembering violent than non-violent events.	47.6	36	No
Eyewitnesses are more accurate when identifying members of their own race than members of other races.	52.8	90	No
An eyewitness’s confidence can be influenced by factors that are unrelated to identification accuracy.	79.5	87	Yes (M_true_ = 2.87, SD = 0.96, M_other_ = 2.54, SD = 0.92, f(486) = 10.033, *p* = 0.002)
Alcoholic intoxication impairs an eyewitness’s later ability to recall persons and events.	83	90	No
Exposure to mug shots of a suspect increases the likelihood that the witness will later choose that suspect in a lineup	69.4	95	No
Traumatic experiences can be repressed for many years and then recovered.	82.3	22	No
Memories people recover from their own childhood are often false or distorted in some way.	51.7	68	No
It is possible to reliably differentiate between true and false memories.	55.6	32	No
Young children are less accurate as witnesses than are adults.	43.9	70	No
Young children are more vulnerable than adults to interviewer suggestion, peer pressures and other social influences.	76.6	94	Yes (M_true_ = 2.86, SD = 0.96, M_other_ = 2.62, SD = 0.87, f(486) = 5.517, *p* = 0.019)
The more members of a lineup resemble a witness’s description of the culprit, the more accurate an identification of the suspect is likely to be.	49.3	71	No
Witnesses are more likely to misidentify someone by making a relative judgement when presented with a simultaneous (as opposed to sequential) lineup. A simultaneous lineup is one where all members in the lineup are presented to thewitness at the same time, and the witness is asked whether the culprit/suspect is in thelineup. In a sequential lineup, the witness is presented each member in the lineup one at atime and the witness is asked whether that person is the culprit/suspect.	55.9	81	No
Elderly eyewitnesses are less accurate than are younger adults.	52.2	50	No
The more quickly a witness makes an identification upon seeing the lineup, the more accurate he or she is likely to be.	66.5	40	No

**Table 3:** Top words not shared between corpora AC and CBC.

AC (39,086 Tokens, 309 Files)			CBC (21,718 Tokens, 178 Files)		
Type	Freq	Range	Type	Freq	Range
wasn	112	74	based	62	38
only	103	68	details	58	41
paying	89	76	identified	54	41
says	87	41	able	43	32
difficult	77	59	assessment	42	28
really	77	60	even	42	31
more	76	54	no	40	31
paid	71	55	believe	39	29
strangers	71	56	seem	38	31
camera	68	58	how	36	26
looked	68	52	much	36	32

Note: “Type” refers to the unique forms of words or phrases, counting each distinct word only once in corpus linguistics. It is distinguished from “token”, which refers to each individual instance of a word or phrase in the corpus. Therefore, tokens represent the total number of words, while types represent the variety of words used. “Range” refers to the number of files in which a word occurs. The contraction for “auxiliary verb + not,” such as “wasn’t” and “doesn’t,” are treated as two separate tokens (e.g., “wasn” + “t,” “doesn” + “t”) in *AntConc*. Therefore, “t” is recognized as a negation marker in this article.

**Table 4:** Top collocates on the right of “says”.

Rank	Collocate	FreqR	Range	Likelihood
1	he	33	19	25.481
2	she	28	19	10.686
3	that	24	16	18.996
4	is	23	17	22.793
4	the	23	14	5.289
6	not	21	19	17.564
7	it	12	11	11.367
8	was	11	11	0.125
9	a	10	9	0.792
10	saw	9	6	8.002
10	difficult	9	9	26.37
10	to	9	8	0.004
13	sure	8	7	3.378
14	good	6	6	1.141
14	confident	6	5	0.056
16	memory	5	5	0.67
16	face	5	3	0.366
16	very	5	4	0.461
16	has	5	5	3.536
16	well	5	4	7.475

**Table 5:** Top collocates on the right side of “t”.

Rank	Collocate	Freq	Range	Likelihood
1	seem	24	22	68.846
2	sure	52	45	50.566
3	paying	25	23	49.183
4	have	32	29	45.038
5	attention	37	33	43.325
6	See	17	17	34.646
7	Any	12	11	33.502
8	At	33	28	28.64
9	witness	8	8	27.694
10	good	40	36	26.537
11	remember	14	12	26.463
12	much	14	14	24.706
13	Not	8	8	24.183
14	Was	15	15	23.095
15	give	9	8	22.275
16	The	97	68	22.11
17	sound	7	6	20.173
18	Pay	10	10	18.953
19	confident	46	39	18.527
20	great	9	7	17.193
21	think	14	14	15.73
22	fully	6	6	15.555
23	Get	7	5	15.248
24	really	13	12	14.85

**Table 6:** Top collocates on both sides of “details” in corpus CBC.

Collocate	FreqLR	FreqL	FreqR	Range	Likelihood
the	58	22	36	30	1.042
and	19	10	9	14	1.053
witness	14	9	5	12	0.464
of	14	4	10	13	0.176
they	13	9	4	9	9.88
specific	13	13	0	9	45.79
to	12	8	4	11	0.009
about	12	1	11	10	16.27
that	12	2	10	11	0.162
person	11	2	9	10	0.174
suspect	10	2	8	6	12.4
she	9	2	7	6	2.478
gave	7	7	0	5	16.997
he	7	3	4	4	5.292
s	7	0	7	5	2.929
on	6	4	2	6	0.684
their	6	5	1	6	1.503
as	6	3	3	5	2.976
face	6	3	3	6	0.459
facial	6	4	2	6	7.765
with	6	1	5	6	1.427
i	6	0	6	5	0.001
was	5	3	2	5	5.992
a	5	3	2	4	2.965
not	5	4	1	5	1.514
provided	5	4	1	4	12.742
matched	5	1	4	4	11.968
confident	5	3	2	4	0.242

## Data Availability

The data from this study are available on OSF at osf.io/7wy2c/.

## Appendix A

**Table A1:** Top 100 keywords in corpus ACBC.

Rank	Type	Freq_Tar	Range_Tar	Keyness (Likelihood)
1	witness	1140	306	6301.6
2	person	927	332	4192.761
3	confident	721	343	3893.981
4	she	1831	367	3476.409
5	identification	433	164	2280.037
6	memory	462	244	2135.866
7	sure	526	293	2005.005
8	faces	399	224	1989.449
9	laptop	339	190	1848.386
10	he	1647	366	1792.416
11	face	508	254	1648.942
12	video	298	183	1430.945
13	confidence	294	142	1398.197
14	saw	398	226	1355.317
15	suspect	267	121	1292.155
16	very	466	266	1218.169
17	good	480	271	1157.195
18	not	964	386	1123.588
19	attention	297	192	1052.662
20	was	1359	405	926.82
21	lineup	146	80	819.119
22	facial	145	104	791.056
23	correct	161	101	706.731
24	remembering	118	84	637.672
25	took	260	155	617.297
26	also	388	213	612.347
27	features	145	104	581.984
28	the	5086	473	555.762
29	unsure	97	87	507.574
30	identify	131	94	504.008
31	strangers	106	76	491.567
32	paying	113	97	459.511
33	seemed	180	121	442.097
34	mentioned	98	63	430.707
35	choice	141	91	425.531
36	identified	114	85	421.374
37	details	115	81	413.689
38	stated	92	62	402.145
39	decision	129	93	400.775
40	clearly	124	97	398.493
41	clear	147	105	371.77
42	pretty	120	97	369.089
43	because	278	162	367.048
44	camera	95	78	332.956
45	wasn	147	94	312.302
46	made	225	140	308.554
47	is	960	342	307.28
48	paid	100	78	307.129
49	admitted	65	45	298.451
49	seems	123	86	296.628
51	thief	59	33	295.572
52	assessment	90	59	289.209
53	description	73	55	285.835
54	accuracy	60	34	274.993
55	t	497	201	271.48
56	difficult	98	75	265.305
56	they	514	139	257.582
58	did	215	147	250.928
59	stole	52	40	245.505
60	able	106	79	238.902
61	identifying	56	40	227.187
62	based	123	81	225.163
63	incorrect	46	33	224.049
64	said	382	199	222.622
65	testimony	53	26	216.667
66	guessing	43	35	215.853
67	defendant	42	29	214.881
68	that	1176	349	213.46
69	guy	83	47	213.079
70	number	131	87	210.386
71	certain	98	76	197.113
72	seem	90	73	197.02
73	believe	102	68	194.899
74	answers	52	47	182.579
75	accurate	50	38	179.893
76	culprit	30	18	172.564
77	uncertainty	44	27	169.584
78	really	110	82	163.531
79	remember	78	64	162.83
80	explanation	43	29	160.804
81	hesitant	28	24	152.476
82	look	113	92	147.377
83	shape	54	46	146.891
84	detail	49	44	145.586
85	picked	54	45	141.077
86	about	298	194	140.435
87	saying	68	59	139.892
88	specific	68	43	139.25
89	doubt	55	42	138.642
90	somewhat	47	37	138.24
91	angle	33	32	136.553
92	didn	135	89	134.857
93	close	81	67	134.459
94	hesitation	28	21	130.067
95	who	310	191	128.851
96	recall	41	33	128.206
97	think	125	93	125.346
98	her	495	233	124.539
99	elimination	26	23	122.742
100	acne	22	18	118.434

**Table A2:** Keywords not shared between corpora AC and CBC.

AC (39,086 Tokens, 309 Files)				CBC (21,718 Tokens, 178 Files)			
Type	Freq_Tar	Range_Tar	Keyness (Likelihood)	Type	Freq_Tar	Range_Tar	Keyness (Likelihood)
t	367	147	264.814	explanation	27	16	137.973
difficult	77	59	242.784	consistent	30	16	112.259
guy	61	32	177.129	matched	24	14	111.666
really	77	60	128.419	shape	29	25	106.333
angle	26	25	122.812	specific	34	20	94.055
didn	100	66	122.368	somewhat	23	18	87.02
picked	40	33	118.579	recollection	12	9	85.84
recall	30	23	105.271	attentive	12	8	77.964
acne	17	14	104.47	videos	13	10	77.142
look	74	61	101.962	individual	31	19	76.301
her	334	159	99.937	hesitation	12	8	70.188
perpetrator	16	7	97.992	likely	34	18	68.179
stranger	25	19	96.162	appeared	24	14	66.558
elimination	18	16	95.006	who	121	71	64.216
think	84	65	91.988	gave	33	24	62.499
close	52	44	89.728	their	131	34	59.38
suspects	18	15	82.969	level	31	17	57.677
picture	36	32	81.66	average	24	19	57.04
lot	50	43	80.641	witnesses	11	10	56.469
judgement	14	13	80.426	match	15	13	55.145
